# The First Step in Standardizing an Artificial Aging Protocol for Dental Composites—Evaluation of Basic Protocols

**DOI:** 10.3390/molecules27113511

**Published:** 2022-05-30

**Authors:** Agata Szczesio-Wlodarczyk, Magdalena Fronczek, Katarzyna Ranoszek-Soliwoda, Jarosław Grobelny, Jerzy Sokolowski, Kinga Bociong

**Affiliations:** 1 University Laboratory of Materials Research, Medical University of Lodz, 92-213 Lodz, Poland; 2 “DynamoLab” Academic Laboratory of Movement and Human Physical Performance, Medical University of Lodz, 92-213 Lodz, Poland; magdalena.fronczek@umed.lodz.pl; 3 Department of Health Sciences, Medical University of Mazovia, 02-091 Warszawa, Poland; 4 Department of Materials Technology and Chemistry, Faculty of Chemistry, University of Lodz, 90-236 Lodz, Poland; katarzyna.soliwoda@chemia.uni.lodz.pl (K.R.-S.); jaroslaw.grobelny@chemia.uni.lodz.pl (J.G.); 5 Department of General Dentistry, Medical University of Lodz, 92-213 Lodz, Poland; jerzy.sokolowski@umed.lodz.pl (J.S.); kinga.bociong@umed.lodz.pl (K.B.)

**Keywords:** resin, composite, dentistry, aging, degradation, clinical performance

## Abstract

The clinical performance of a dental restoration is strongly influenced by the complex and dynamically-changing oral environment; however, no standard procedure exists to evaluate this lifetime. This research provides an in-depth analysis of the effect of different aging procedures on the flexural strength (FS), diametral tensile strength (DTS) and hardness (HV) of selected dental materials (Resin F, Flow-Art and Arkon). Material structure was evaluated by scanning electron microscopy. It was found that each aging protocol had some influence on the tested properties, with continual erosion and degradation being observed. Greater mechanical degradation was observed for Resin F (neat resin) after the applied aging protocols, suggesting that a resin matrix is more susceptible for degradation. The most aggressive aging protocol was Protocol 5: 0.1 M NaOH, seven days, 60 °C. Further studies on the effect of artificial aging on dental materials should include a study of the thermal and chemical factors. A standardized aging procedure is crucial for improving the resistance of dental resin composite to oral conditions and their clinical performance.

## 1. Introduction

The most commonly-used materials in restorative dentistry are resin composites [1,2]. However, such resins are subject to degradation, given the complex nature of the oral environment [3], for example, saliva, food and drink, biofilm, varying temperature, wear processes and intraoral loads can all adversely affect clinical performance [4,5,6,7,8]. Eventually, due to the progressive degradation of the composite material and bonding system, all dental restorations will need to be replaced [9]. 

However, some materials are more resistant to degradation than others, and the resistance of such composites to various aging processes has been evaluated in a number of in vitro studies. Generally, these studies evaluate changes in properties of dental materials under selected aging conditions [10,11,12,13,14]. Material aging is typically indicated by leaching of degradation products and unreacted substrates, reduced strength, and hardness, increased roughness, increased abrasiveness, color change, increasing water sorption and cracking [15]. The most important factor influencing the degradation processes is material composition (resin matrix, filler and coupling agent) [16,17,18], and the most commonly-used solvents in dental material research are water, artificial saliva, ethanol, NaOH solution, food and beverages [19,20,21,22,23]. 

Given the complexity of the oral environment, aging tests should not be performed using only water as aging solution. Instead, more aggressive environments using ethanol and NaOH solutions are used, as these are believed to accelerate the hydrolysis process [11,14]. Chemical degradation is the first important aspect in evaluation of dental composites clinical performance. The second is a simulation of dynamically changing oral environment. Thermocycling can be used to imitate the temperature fluctuations which occur in the oral environment; such changes can result in intensive aging, possibly due to the occurrence of internal stress caused by dimensional changes of the resin and fillers, which have different thermal expansion coefficients [24,25]. A study of the artificial aging of dental cements found the most efficient thermal cycling protocol to be (5 °C/55 °C/1 min) for four days; however, it was also found that storage in water for four days at 55 °C may be considered a viable alternative to thermal cycling [26].

So far, no attempt has been made to design a standard protocol to estimate dental composite clinical performance in a practical and effective way. Dental materials do not have to undergo research to validate their performance over their predicted lifetime upon introduction to the market. ISO 4049 specifies only the use of sorption and solubility tests in a water environment. High sorption and solubility values can weaken the restoration [27]. However, these studies do not provide any information on how the material will behave during prolonged use in a complex oral environment. There is a clear need to identify procedures that will accurately replicate the oral environment and can be used to determine the aging process of dental materials [15]. A standardized aging procedure is crucial for improving the resistance of dental resin composite to oral conditions. In addition, the use of a wider and more accurate selection of factors and research methods during artificial aging protocols will provide a clearer picture of the prolonged clinical performance of dental composites.

Therefore, the aim of the study is to select preliminary protocols (maximum three) for determining composite degradation, based on an evaluation of chosen aging procedures. In this study three materials were tested: neat dental resin and two composites (flow and universal) based on a similar resin matrix. 

## 2. Results

The most popular research methods in evaluation of dental composites were selected for this study. The three-point bending flexural strength (FS), diametral tensile strength (DTS) and Vickers hardness (HV) were determined. Sorption and solubility of the tested materials were established according to the ISO 4049 standard. In addition, chemical composition analysis and microstructure evaluation of tested samples was conducted using energy dispersive spectroscopy and scanning electron microscopy (SEM).

**Figure A1:**
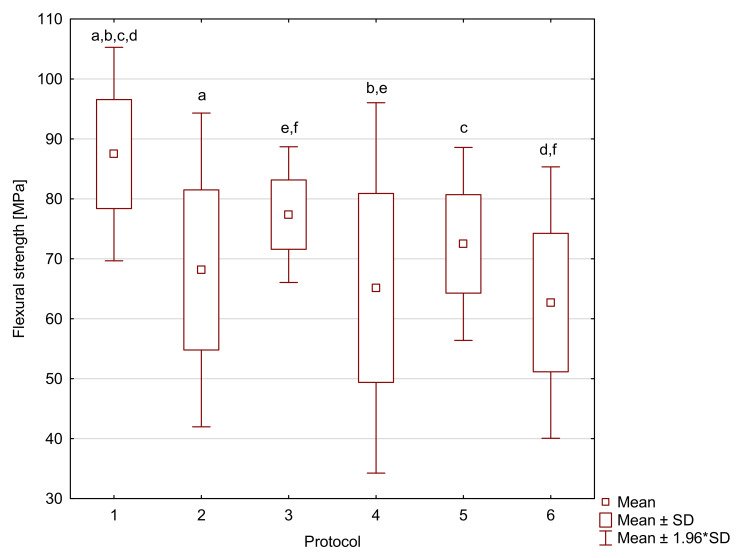
Box-and-whisker plot of three-point bending flexural strength (FS) of Resin F material. Statistically-significant differences were detected between the following: (a,b,c,d) Protocol 1 (water, 24 h, 37 °C) vs. (a) Protocol 2 (artificial saliva, 90 days, 37 °C) (*p* = 0.002517); (b) Protocol 4 (water, 7500 thermocycles, 5/55 °C) (*p* = 0.000617); (c) Protocol 5 (0.1 M NaOH, 7 days, 60 °C) (*p* = 0.016375); (d) Protocol 6 (water, 5 days, 55 °C) (*p* = 0.000187); (e,f) Protocol 3 (water, 500 thermocycles, 5/55 °C) vs. (e) Protocol 4 (water, 7500 thermocycles, 5/55 °C) (*p* = 0.047185); (f) Protocol 6 (water, 5 days, 55 °C) (*p* = 0.018579).

**Figure A2:**
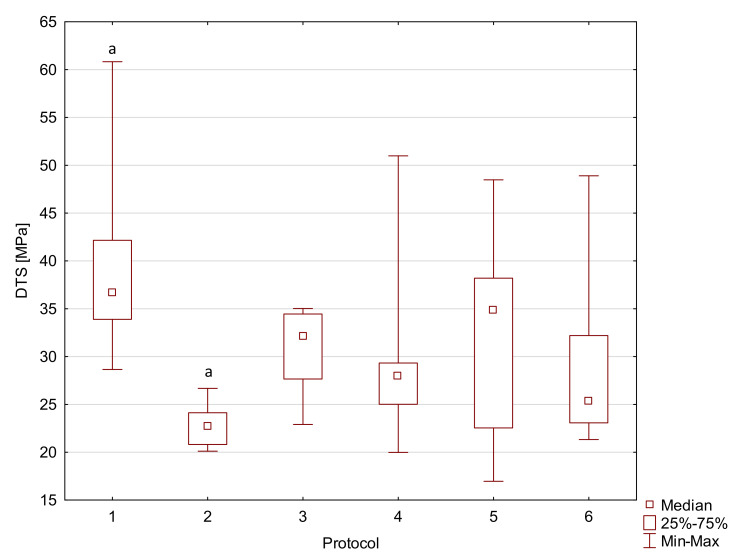
Box-and-whisker plot of diametral tensile strength (DTS)) of Resin F material. Statistically-significant differences were detected between the following: (a) Protocol 1 (water, 24 h, 37 °C) vs. (a) Protocol 2 (artificial saliva, 90 days, 37 °C) (*p* = 0.000532).

**Figure A3:**
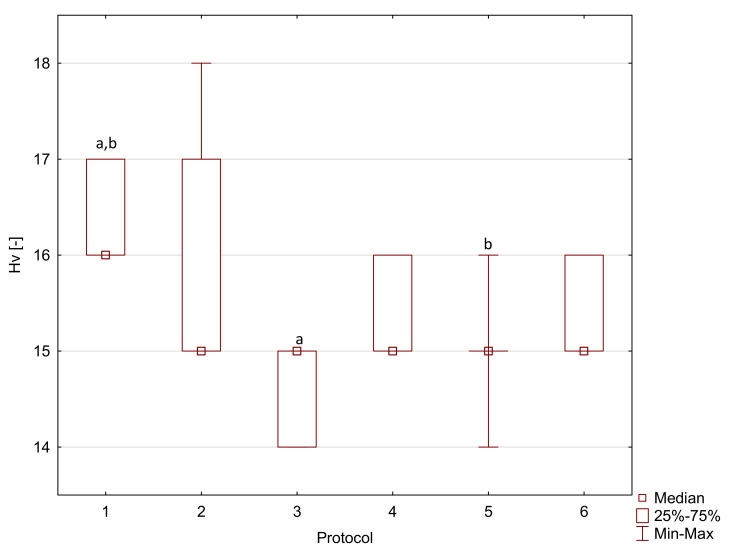
Box-and-whisker plot of Vickers hardness (HV) of Resin F material. Statistically-significant differences were detected between the following: (a,b) Protocol 1 (water, 24 h, 37 °C) vs. (a) Protocol 3 (water, 500 thermocycles, 5/55 °C) (*p* = 0.000314); (b) Protocol 5 (0.1 M NaOH, 7 days, 60 °C) (*p* = 0.024805).

**Figure A4:**
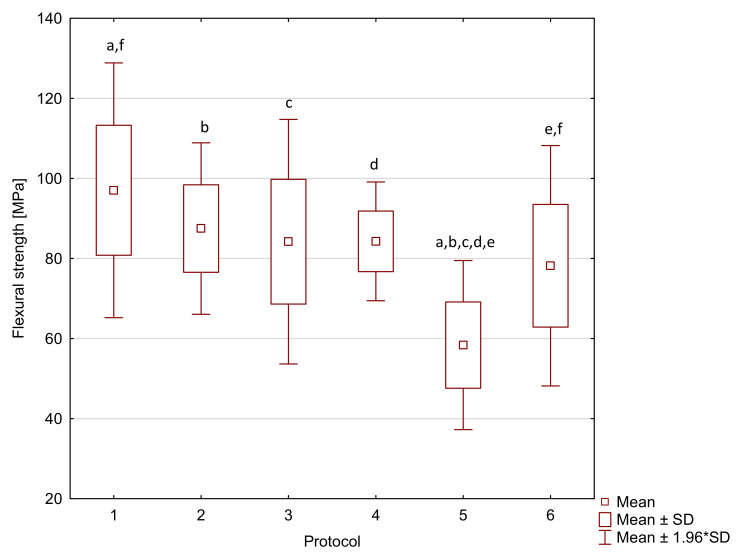
Box-and-whisker plot of three-point bending flexural strength (FS) of Flow-Art material. Statistically-significant differences were detected between the following: (a,b,c,d,e) Protocol 5 (0.1 M NaOH, 7 days, 60 °C) vs. (a) Protocol 1 (water, 24 h, 37 °C) (*p* = 0.000003); (b) Protocol 2 (artificial saliva, 90 days, 37 °C) (*p* = 0.000194); (c) Protocol 3 (water, 500 thermocycles, 5/55 °C) (*p* = 0.000756); (d) Protocol 4 (water, 7500 thermocycles, 5/55 °C) (*p* = 0.000730); (e) Protocol 6 (water, 5 days, 55 °C) (*p* = 0.007684); (f) Protocol 6 (water, 5 days, 55 °C) vs. (f) Protocol 1 (water, 24 h, 37 °C) (*p* = 0.010828).

**Figure A5:**
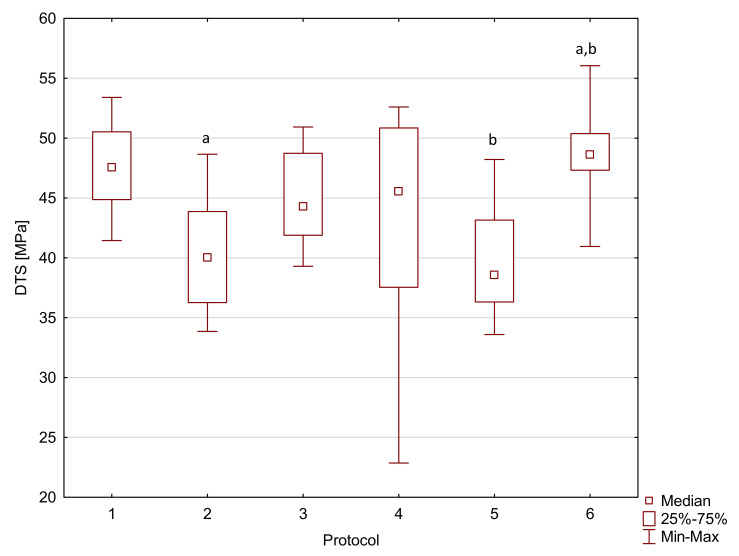
Box-and-whisker plot of diametral tensile strength (DTS) of Flow-Art material. Statistically-significant differences were detected between the following: (a,b) Protocol 6 (water, 5 days, 55 °C) vs. (a) Protocol 2 (artificial saliva, 90 days, 37 °C) (*p* = 0.028899); (b) Protocol 5 (0.1 M NaOH, 7 days, 60 °C) (*p* = 0.026107).

**Figure A6:**
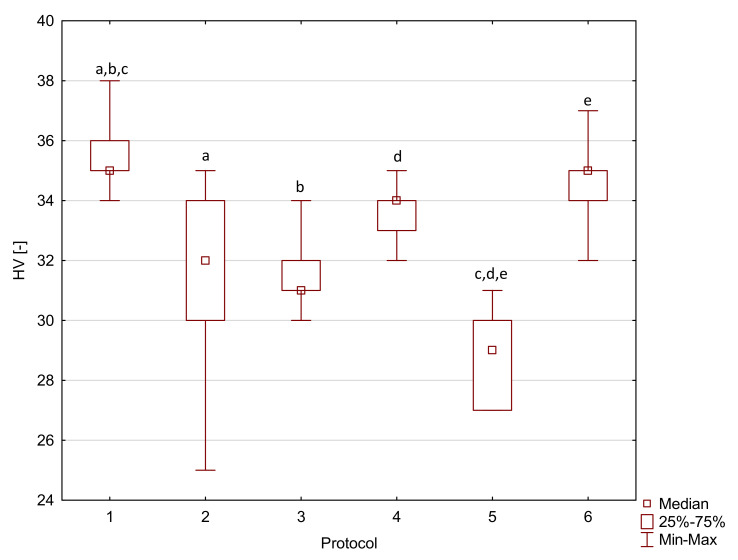
Box-and-whisker plot of Vickers hardness (HV) of Flow-Art material. Statistically-significant differences were detected between the following: (a,b,c) Protocol 1 (water, 24 h, 37 °C) vs. (a) Protocol 2 (artificial saliva, 90 days, 37 °C) (*p* = 0.010352); (b) Protocol 3 (water, 500 thermocycles, 5/55 °C) (*p* = 0.004079); (c) Protocol 5 (0.1 M NaOH, 7 days, 60 °C) (*p* = 0.000003); (d,e) Protocol 5 (0.1 M NaOH, 7 days, 60 °C) vs. (d) Protocol 4 (water, 7500 thermocycles, 5/55 °C) (*p* = 0.016355); (e) Protocol 6 (water, 5 days, 55 °C) (*p* = 0.000164).

**Figure A7:**
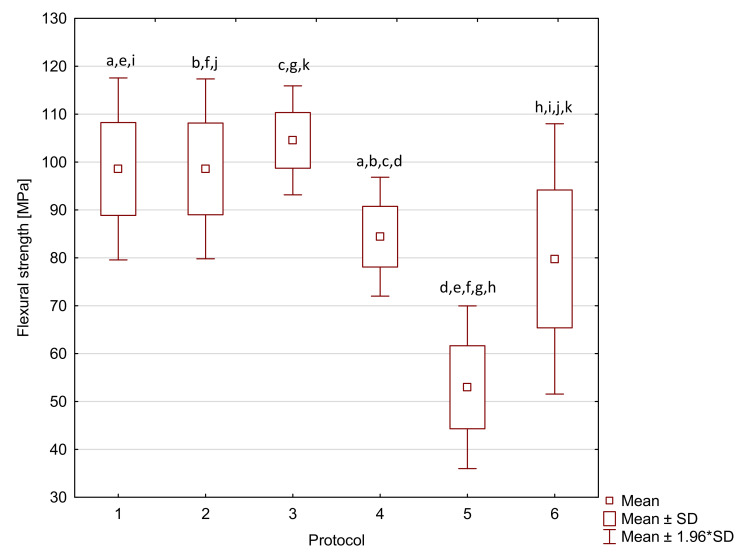
Box-and-whisker plot of three-point bending flexural strength (FS) of Arkon material. Statistically-significant differences were detected between the following: (a,b,c,d) Protocol 4 (water, 7500 thermocycles, 5/55 °C) vs. (a) Protocol 1 (water, 24 h, 37 °C) (*p* = 0.008509); (b) Protocol 2 (artificial saliva, 90 days, 37 °C) (*p* = 0.008449); (c) Protocol 3 (water, 500 thermocycles, 5/55 °C) (*p* = 0.000340); (d) Protocol 5 (0.1 M NaOH, 7 days, 60 °C) (*p* = 0.000000); (e,f,g,h) Protocol 5 (0.1 M NaOH, 7 days, 60 °C) vs. (e) Protocol 1 (water, 24 h, 37 °C) (*p* = 0.000000); (f) Protocol 2 (artificial saliva, 90 days, 37 °C) (*p* = 0.000000); (g) Protocol 3 (water, 500 thermocycles, 5/55 °C) (*p* = 0.000000); (h) Protocol 6 (water, 5 days, 55 °C) (*p* = 0.000006); (i,j,k) Protocol 6 (water, 5 days, 55 °C) vs. (i) Protocol 1 (water, 24 h, 37 °C) (*p* = 0.000721); (j) Protocol 2 (artificial saliva, 90 days, 37 °C) (*p* = 0.000716); (k) Protocol 3 (water, 500 thermocycles, 5/55 °C) (*p* = 0.000022).

**Figure A8:**
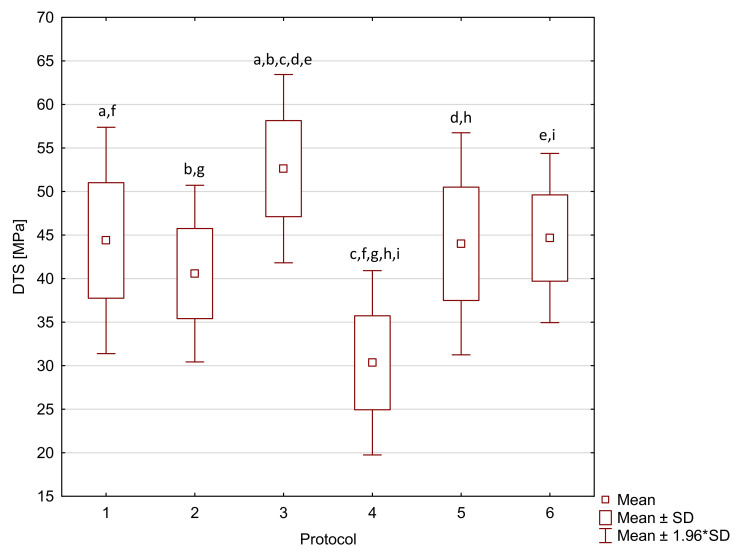
Box-and-whisker plot of diametral tensile strength (DTS) of Arkon material. Statistically-significant differences were detected between the following: (a,b,c,d,e) Protocol 3 (water, 500 thermocycles, 5/55 °C) vs. (a) Protocol 1 (water, 24 h, 37 °C) (*p* = 0.003699); (b) Protocol 2 (artificial saliva, 90 days, 37 °C) (*p* = 0.000049);(c) Protocol 4 (water, 7500 thermocycles, 5/55 °C) (*p* = 0.00000); (d) Protocol 5 (0.1 M NaOH, 7 days, 60 °C) (*p* = 0.002486); (e) Protocol 6 (water, 5 days, 55 °C) (*p* = 0.004921); (f,g,h,i) Protocol 4 (water, 7500 thermocycles, 5/55 °C) vs. (f) Protocol 1 (water, 24 h, 37 °C) (*p* = 0.000004); (g) Protocol 2 (artificial saliva, 90 days, 37 °C) (*p* = 0.000422); (h) Protocol 5 (0.1 M NaOH, 7 days, 60 °C) (*p* = 0.000007); (i) Protocol 6 (water, 5 days, 55 °C) (*p* = 0.000003).

**Figure A9:**
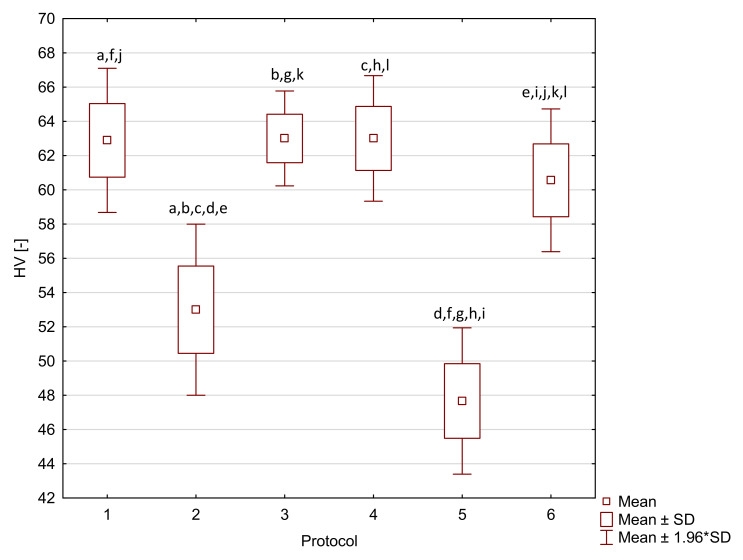
Box-and-whisker plot of Vickers hardness (HV) of Arkon material. Statistically-significant differences were detected between the following: (a,b,c,d,e) Protocol 2 (artificial saliva, 90 days, 37 °C) vs. (a) Protocol 1 (water, 24 h, 37 °C) (*p* = 0.000000); (b) Protocol 3 (water, 500 thermocycles, 5/55 °C) (*p* = 0.000000); (c) Protocol 4 (water, 7500 thermocycles, 5/55 °C) (*p* = 0.000000); (d) Protocol 5 (0.1 M NaOH, 7 days, 60 °C) (*p* = 0.000002); (e) Protocol 6 (water, 5 days, 55 °C) (*p* = 0.000000); (f,g,h,i) Protocol 5 (0.1 M NaOH, 7 days, 60 °C) vs. (f) Protocol 1 (water, 24 h, 37 °C) (*p* = 0.000000); (g) Protocol 3 (water, 500 thermocycles, 5/55 °C) (*p* = 0.000000); (h) Protocol 4 (water, 7500 thermocycles, 5/55 °C) (*p* = 0.000000); (i) Protocol 6 (water, 5 days, 55 °C) (*p* = 0.000000); (j,k,l) Protocol 6 (water, 5 days, 55 °C) vs. (j) Protocol 1 (water, 24 h, 37 °C) (*p* = 0.021185); Protocol 3 (water, 500 thermocycles, 5/55 °C) (*p* = 0.016035); (l) Protocol 4 (water, 7500 thermocycles, 5/55 °C) (*p* = 0.016035).

The first part of this study evaluated a number of selected basic protocols based on a literature review [15]. The obtained flexural strength, diametral tensile strength and hardness values of the tested materials were presented in Table 1. Box-and-whisker plots of the obtained results and exact *p*-values are shared in Appendix A (Figure A1, Figure A2, Figure A3, Figure A4, Figure A5, Figure A6, Figure A7, Figure A8 and Figure A9). Almost all samples demonstrated degradation in FS, DTS and HV values after using aging protocols compared to the control group, i.e., those subjected to Protocol 1. 

The sorption and solubility of the used materials are presented in Table 2. The highest sorption and solubility values were observed for Resin F, and the lowest for Arkon material.

**Figure 1:**
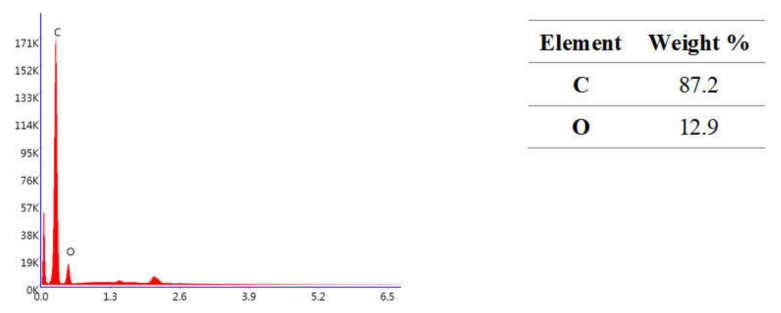
Chemical composition analysis of Resin F material after Protocol 1 (24 h, 37 °C, distilled water). Data were obtained by energy dispersive spectroscopy (EDS).

**Figure 2:**
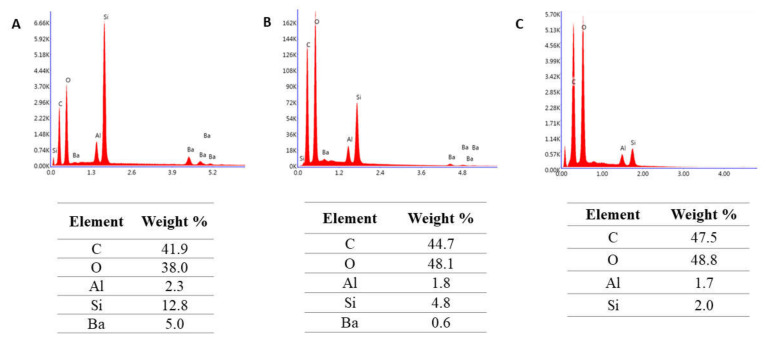
Chemical composition analysis of Flow-Art material after (**A**) Protocol 1 (24 h, 37 °C, distilled water), (**B**) Protocol 2 (three months, 37 °C, artificial saliva), (**C**) after Protocol 5 (1 week, 60 °C, 0.1 M NaOH). Data were obtained by energy dispersive spectroscopy (EDS).

**Figure 3:**
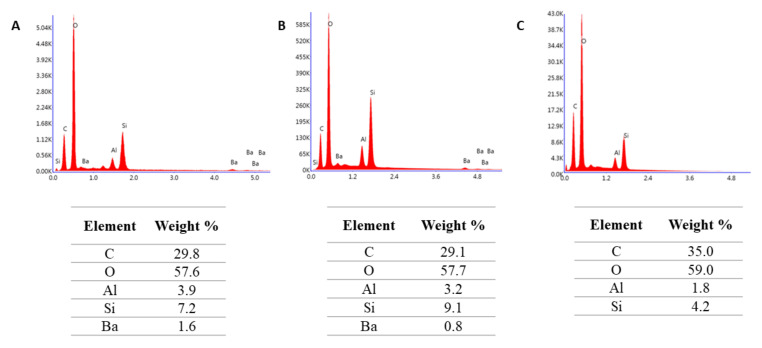
Chemical composition analysis of Arkon material after (**A**) Protocol 1 (24 h, 37 °C, distilled water), (**B**) Protocol 2 (three months, 37 °C, artificial saliva), (**C**) after Protocol 5 (1 week, 60 °C, 0.1 M NaOH). Data were obtained by energy dispersive spectroscopy (EDS).

The selected results of the chemical composition analysis of tested materials are given in Figure 1, Figure 2 and Figure 3. Resin F is composed of the resin itself and only the signals corresponding to carbon and oxygen are visible in the analyzed spectra (Figure 1). In contrast, elements from the fillers can also be seen in the Flow-Art and Arkon materials: aluminum (Al), silicon (Si) and barium (Ba) (Figure 2 and Figure 3).

**Figure 4:**
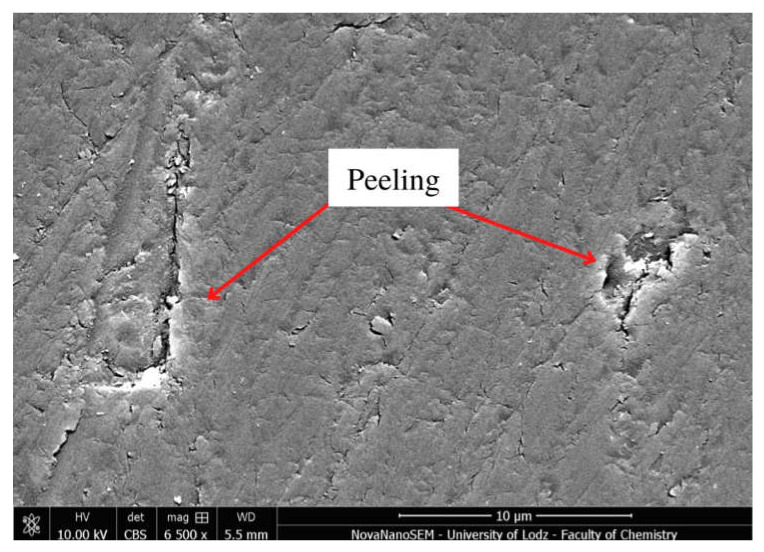
Scanning electron microscopy (SEM) micrograph of Resin F at 6500× magnification after Protocol 5 (one week, 60 °C, 0.1 M NaOH).

**Figure 5:**
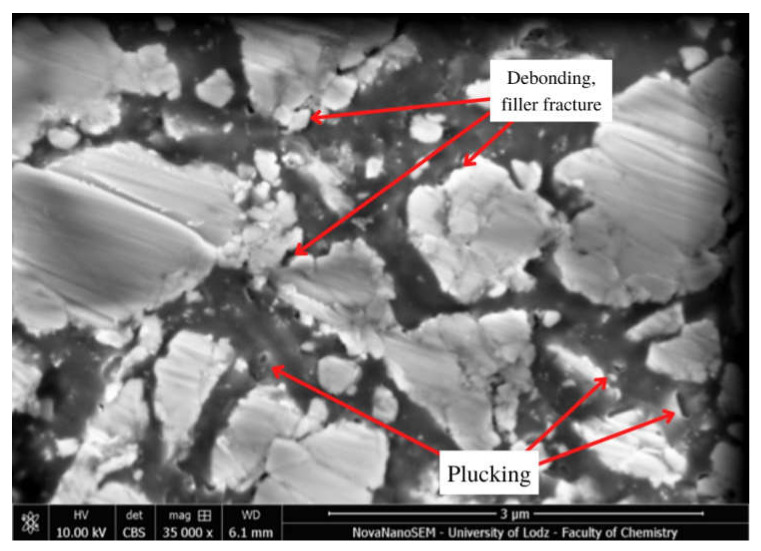
Scanning electron microscopy (SEM) micrograph of Flow-Art at 35,000× magnification after Protocol 5 (one week, 60 °C, 0.1 M NaOH).

**Figure 6:**
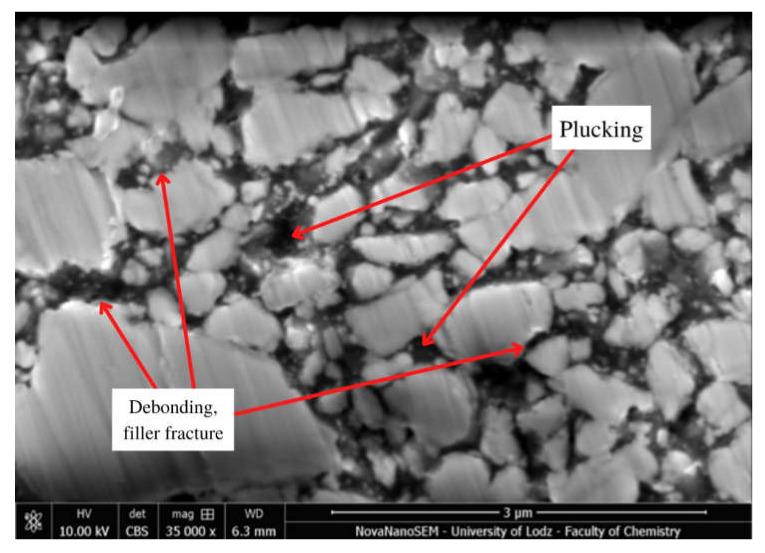
Scanning electron microscopy (SEM) micrograph of Arkon at 35,000× magnification after Protocol 5 (one week, 60 °C, 0.1 M NaOH).

Selected SEM micrographs showing surface degradation in Resin F, Flow-Art and Arkon materials after Protocol 5 (one week, 60 °C, 0.1 M NaOH) are presented in Figure 4, Figure 5 and Figure 6. 

**Figure A10:**
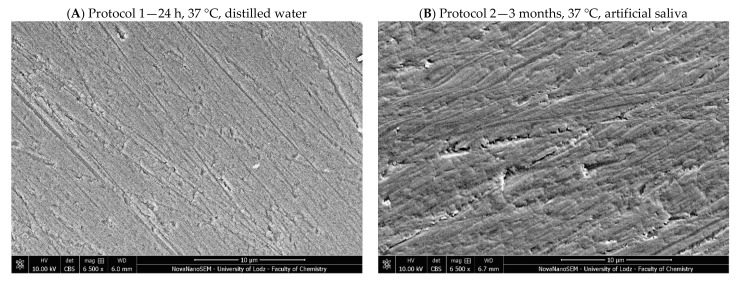

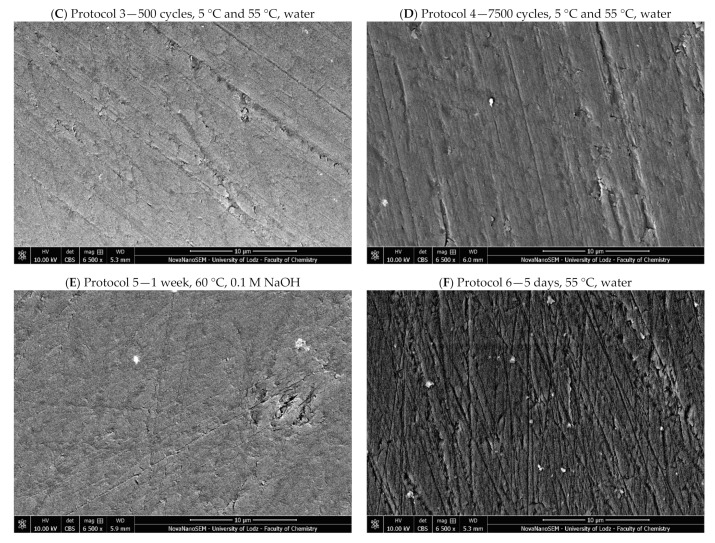
Scanning electron microscopy (SEM) micrograph of Resin F at 6500× magnification after six aging protocols: (**A**) Protocol 1—24 h, 37 °C, distilled water, (**B**) Protocol 2—3 months, 37 °C, artificial saliva, (**C**) Protocol 3—500 cycles, 5 °C and 55 °C, water, (**D**) Protocol 4—7500 cycles, 5 °C and 55 °C, water, (**E**) Protocol 5—1 week, 60 °C, 0.1 M NaOH, (**F**) Protocol 6—5 days, 55 °C, water.

**Figure A11:**
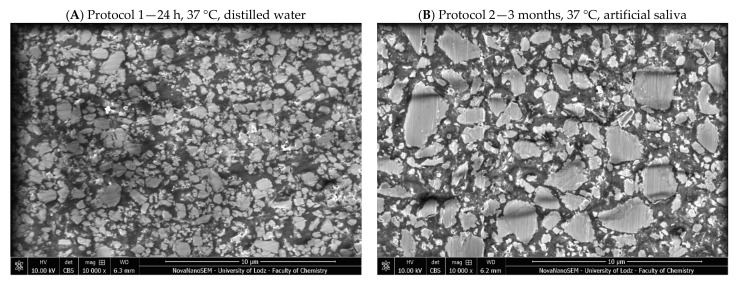

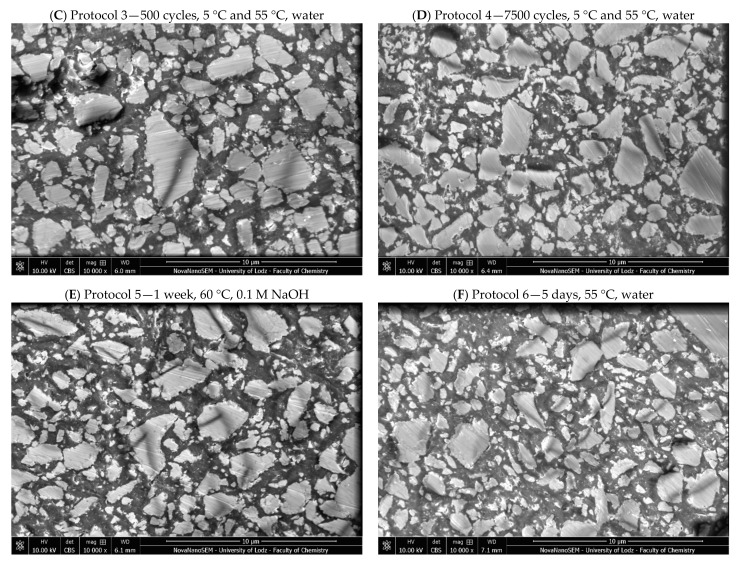
Scanning electron microscopy (SEM) micrograph of Flow-Art at 10,000× magnification after six aging protocols: (**A**) Protocol 1—24 h, 37 °C, distilled water, (**B**) Protocol 2—3 months, 37 °C, artificial saliva, (**C**) Protocol 3—500 cycles, 5 °C and 55 °C, water, (**D**) Protocol 4—7500 cycles, 5 °C and 55 °C, water, (**E**) Protocol 5—1 week, 60 °C, 0.1 M NaOH, (**F**) Protocol 6—5 days, 55 °C, water.

**Figure A12:**
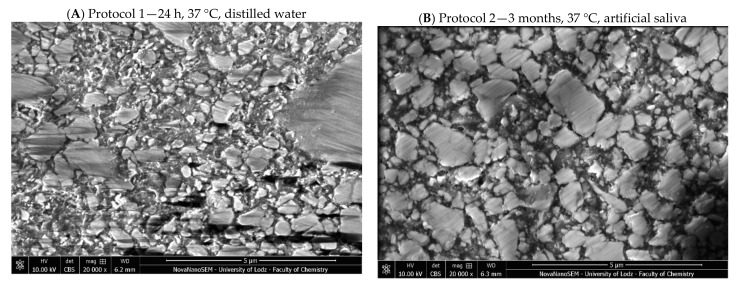

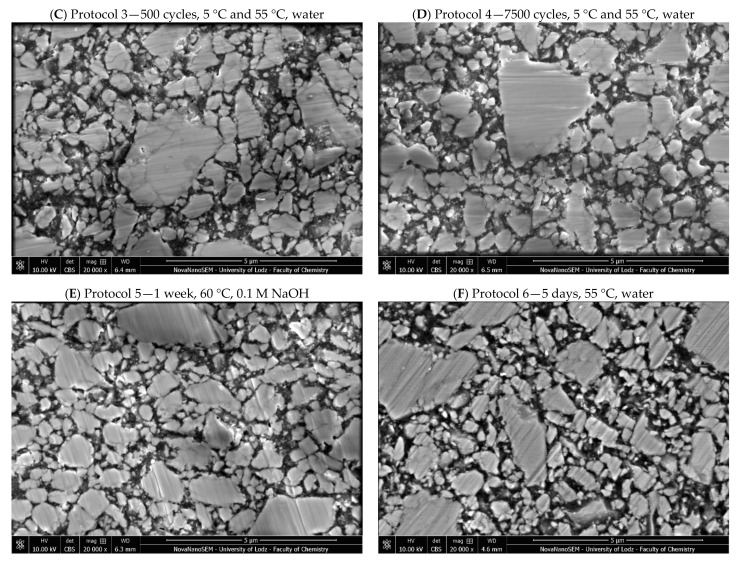
Scanning electron microscopy (SEM) micrograph of Arkon at 20,000× magnification after six aging protocols: (**A**) Protocol 1—24 h, 37 °C, distilled water, (**B**) Protocol 2—3 months, 37 °C, artificial saliva, (**C**) Protocol 3—500 cycles, 5 °C and 55 °C, water, (**D**) Protocol 4—7500 cycles, 5 °C and 55 °C, water, (**E**) Protocol 5—1 week, 60 °C, 0.1 M NaOH, (**F**) Protocol 6—5 days, 55 °C, water.

This aging process—Protocol 5 (one week, 60 °C, 0.1 M NaOH)—resulted in the greatest changes in surface structure among the tested protocols. There are visible places where the filler was rinsed out (plucking) or fractured, the connection between the matrix and the filler was broken (debonding), or where the polymer matrix was delaminated (peeling). The observed changes were also visible after the use of other protocols, particularly Protocol 2, 4 and 6 (Figure A10, Figure A11 and Figure A12), indicating the ongoing erosion and degradation of the tested materials.

## 3. Discussion

Unfortunately, despite continuous improvements, dental restorations still suffer from limited fracture resistance, and wear, a lack of consistent degree of conversion, and polymerization shrinkage stress [28]. Using dental composites also involves the risk of debonding. The degradation of connection between the dentin and the composite material with bond, the so-called hybrid layer, is also subject to degradation. The aging processes of this layer cause micro leakage, dentin hyper-sensitivity, marginal pigmentation and may lead to the failure of dental restorations [29,30]. Indeed, composite aging is influenced by a range of chemical, physical and mechanical processes, which may interact with each other depending environmental factors such as the chemical characteristics, mechanical load and wear processes, and on the characteristics of the material [31]. These interactions may accelerate the degradation of resin matrix, fillers and coupling agent. Many studies have been performed to develop more accurate and flawless dental reconstruction materials; as such, there is a need to establish a standard procedure for assessing the clinical performance of dental composites.

Therefore, the present study compared the effects of six existing protocols, as part of an initial attempt to standardize the aging protocol for dental materials. These protocols were selected based on a systematic review [15]. Three of the most commonly-assessed variables, viz. flexural strength, diametral tensile strength and hardness, were selected for the assessment; in addition, the microstructures of the materials were also evaluated by scanning electron microscopy. From the obtained results, it can be seen that the tested properties of Resin F change more drastically after each selected aging protocols compared to the two filled materials (Flow-Art, Arkon). The largest differences, some of them were statistically significant, for mechanical strength (FS, DTS) were observed between the resin material control group and after 2,4,6 aging protocols (Protocol 2—Artificial saliva, 90 days, 37 °C; water, Protocol 4—7500 thermocycles, 5/55 °C; Protocol 6—Water, 5 days, 55 °C).

The tested materials are based on conventional monomers such as Bis-GMA, TEGDMA, UDMA, Bis-EMA. Such polymer networks are characterized by esters, urethanes, amides, hydrogen bonds, and van der Waals interactions [32]. The polymer matrix is also subject to chemical hydrolysis due to the presence of water in the dental composite, and it depends on the type of bond; for example, esters are one of the most susceptible bonds to nucleophilic attack by water [7]. Water sorption is also influenced by monomer type. It is believed that in polymers, the diffusion process can take place either by water entering the polymer material through nanopores and other voids in the polymer material without any chemical reactions (free volume theory), or by diffusing through the material by chemical interactions with the polymer matrix (interaction theory) [33]. Functional groups such as urethanes and hydroxyls, which occur in UDMA and Bis-GMA monomers respectively, may bind water molecules [34]. Ethylene oxide linkages that are present in TEGDMA can also easily accept hydrogen bonds. Due to the occurrence of two phenyl rings, Bis-EMA is the more hydrophobic molecule, despite the presence of ethoxylated groups [34]. The monomers constituting the matrix of the tested materials demonstrate the following water sorption values: 51.2 µg/mm^3^ (Bis-GMA), 42.3 µg/mm^3^ (UDMA), 28.8 µg/mm^3^ (TEGDMA) and 21.3 µg/mm^3^ (Bis-EMA) [35]. 

The highest water sorption values were observed for neat resin material (Table 2). This greater volume of water penetrating into the unfilled polymer network will significantly affect the rate of degradation. This hydrolytic degradation can also be accelerated by the presence of acids, bases and enzymes, particularly bacterial enzymes, and oral physiology [15]. Aging in water alone, either at higher or variable temperatures, accelerates the deterioration of resin (Table 1), with water sorption by resin, leading to plasticization and swelling of the polymer matrix. The progressive hydrolysis of resins results in further swelling, thus allowing easier diffusion of unreacted monomers and degradation products out of the materials [17,36]. Artificial saliva also influenced the hardness of all tested materials, resulting in the swelling, softening and degradation of the polymer matrix; this protocol also had a greater effect on the strength properties of Resin F and Flow-Art compared to water.

All of the aging protocols had a less visible effect on the filled materials compared to the Resin F samples; this could be due to the smaller amount of resin in the composite materials: Flow-Art has about 37% wt. polymer matrix and Arkon materials about 20% wt. The observed changes for Resin F may result not only from greater degradation of the polymer matrix, but also from changes in the internal polymer structure of individual materials. The resin forms the matrix of the composite material, binding the individual filler particles together through a coupling agent [37]. The water responsible for the degradation processes penetrates not only through the polymeric phase by diffusion, but also via the interface between the fillers and the polymer matrix. It has been found that the addition of a coupling agent initially reduced hydrolytic degradation; however, the coupling agent can itself undergo hydrolysis, opening up an extra pathway for water diffusion. Its hydrolytic stability in a complex oral environment is an area of concern [38,39,40]. 

Protocol 5 (0.1 M NaOH, 7 days, 60 °C) significantly decreased the flexural strength and hardness of both composites, but seems to have no observable effect on Resin F. Most likely, the NaOH solution accelerates coupling agent degradation. It was showed that degradation of the interface results in fillers debonding, leaching of ingredients, micro cracks and the reduction of mechanical properties [15,18,41].

Protocol 4 (7500 thermocycles, 5/55 °C) resulted in significant changes in the strength properties (FS, DTS) for Arkon material, which is characterized by a high filler content. No such changes were observed after only 500 thermocycles (Protocol 3); hence, significant differences were observed between Protocols 3 and 4. This is in accordance with previous findings indicating that changes in FS and HV occur after using more than 4000 thermocycles [42]. The significantly greater effects of thermocycling demonstrated on Arkon material compared to Flow-Art and Resin F may be due to the high share of interface between the resin and the filler, where internal stress may occur. Stress can be generated due to dimensional changes of resin and fillers, which have different thermal expansion coefficients [24,25]. Additionally, apart from silica, two types of glass are present in the tested material.

The elemental composition spectra (Figure 2 and Figure 3) indicated that the tested composite materials Flow-Art and Arkon demonstrate a reduction in the amount of barium and silica after more aggressive protocols. This suggests that fillers are dissolved during the aging processes. Indeed, glasses, particularly radiopaque ones, are known to be more susceptible to dissolution in water solutions than silica or quartz [3,43,44]. Hydrolysis of the siloxane bonds causes the formation of hydroxide ions, which can further break the silane–filler interface. The resulting silanol groups cause the formation of a negative charge, which may slow down the hydrolysis; however, this effect is balanced by the positive ions present in the oral environment, which can neutralize the charges and cause progressive degradation of siloxane bonds. Due to the positive ions in artificial salvia, greater loss of filler is observed in this media than in distilled water [45]. This degradation is accelerated by the leaching out of the filler, which creates new pores and voids; these weaken the material microstructure and filler/matrix interface and lead to a more rapid deterioration of the restoration [46,47]. 

Some changes in bulk microstructure are observed (Figure 4, Figure 5 and Figure 6) for all tested aging protocols even those less aggressive ones (Protocol 3 and 6). In such environments, the residual monomers and less bound filler particles are first washed away from the surface of the material. Following this, more progressive degradation occurs of the resin, fillers and coupling agent, which causes greater changes in the microstructure. The SEM analysis reveals the presence of areas from which filler particles seem to have been debonded and plucked out (Figure 5 and Figure 6) in the examined composite materials. This was particularly visible for Protocol 4, i.e., aging in NaOH and 7500 thermocycles, where exposed particles and loosening of filler particles were observed on the surface. It seems that macrofillers demonstrate an additional fracture after chemical and thermal aging, which may suggest further degradation processes. The ongoing degradation could also be observed in the case of the resin itself (Figure A3). Only Protocol 3 had no apparent effect on the observed microstructure, with the surface being smother compared to the other aging methods. Some peeling of the top layer may be noticed when chemical aging (NaOH, Protocol 5) is used (Figure 4). 

These findings suggest that progressive degradation weakens composite microstructures, especially the filler/matrix interface, with the glass fillers being leached and creating new voids; these voids allow further loss of degradation products. In addition, the acid–base functionality of the degradation products may influence the pH inside the polymer network structure, accelerating the hydrolysis processes [41]. As indicated by the Griffith theory of fracture, any type of defect in materials microstructure can act as a crack for brittle materials such as dental composites [48].

On the basis of the obtained data, three protocols were selected for the second part of the study (standardization of artificial aging protocol for dental composites). The first was Protocol 5, NaOH solution, with this being one of the most aggressive protocols used in the study. The second was a combination of Protocol 4 (7500 thermocycles) followed by Protocol 5. Finally, the third protocol was a combination of Protocol 6 followed by Protocol 5. When using Protocol 6, the properties did not change as drastically as with Protocols 4 and 5; however, the protocol still significantly affects the properties of Resin F and may be an alternative procedure for studies without specialized equipment needed to perform thermocycling. The hydrolysis of resin-based dental composites involves interaction with an OH^−^ ions. Compared to water or saliva (pH around 7), 0.1 M NaOH solution (pH = 13) has one million more hydroxyl ions [23]. Chemical reactions and diffusion can also be accelerated by an increased temperature. Taking this into account and knowing that such a prediction is complex it can be assumed that the proposed aging protocols will simulate several years in oral environment.

In the next stage, it is planned to supplement the basic tests (FS, DTS, HV) for selected protocols (Protocol 5, Protocol 4 + 5; Protocol 6 + 5) with fatigue evaluation. Dynamic tests better mimic clinical conditions and can be very valuable in predicting the clinical performance of materials [49,50]. The final stage of standardizing an artificial aging protocol for dental composites assumes the evaluation of selected preliminary protocols on a larger group of dental composite materials. We hope to determine an artificial aging method for evaluating the clinical long term performance of dental composites.

## 4. Materials and Methods

All three studied materials were from Arkona (Niemce, Poland). The first material (Resin F) was unfilled resin. The second and third materials were composites based on a similar resin matrix to Resin F but with differentiated fillers. By examining the behavior of the polymer matrix under the influence of the aging protocols, the stability of the matrix can be assessed in comparison to the stability of the filler system and the filler-matrix interface. A more complete description of tested materials is given in Table 3.

Samples were prepared using silicone molds. In order to avoid the formation of an inhibition layer, the surfaces of the materials were covered with a polyester tape (Hawe Striproll, Kerr, Bioggo, Switzerland). Direct contact of optical fiber with the sample surface was ensured. Specimens were polymerized using LED curing light with 1250 mW/cm^2^ light irradiance (Optilux (the CURE—TC-01, Spring Health Products, Norristown, PA, USA). Curing time was consistent with the manufacturer’s instructions (Table 3).

### 4.1. Aging Protocols

Six basic conditioning protocols were selected to this research (Table 4). 

### 4.2. Methods

The influence of each aging protocol on the material properties of the samples was determined based on flexural strength (FS), diametral tensile strength (DTS), Vickers hardness (HV) and microstructure evaluation. 

#### 4.2.1. Flexural Strength 

Flexural strength (FS) was determined using the three-point bending test. Rectangular samples (dimensions: 2 mm × 2 mm × 25 mm) were used for the tests. For each study group, seven samples were tested. Measurements were carried out using a Zwick Roell Z020 universal testing machine (Zwick–Roell, Ulm, Germany). The traverse speed was 1 mm/min.

#### 4.2.2. Diametral Tensile Strength

The diametral tensile strength (DTS) was performed on cylindrical samples (6 mm in diameter and 3 mm in height). Nine samples were tested from each study group, with all measurements performed using a Zwick Roell Z020 universal testing machine (Zwick–Roell, Ulm, Germany). The traverse speed was 2 mm/min.

#### 4.2.3. Hardness

The hardness of tested materials were measured based on the Vickers method using a Zwick ZHVμm hardness tester (Zwick–Roell, Ulm, Germany). The applied load was 1000 g and the penetration time 10 s. Nine measurements were performed on three out of the nine DTS samples for each study group.

#### 4.2.4. Water Sorption and Solubility

The ISO 4049 standard was used to determine the solubility value [27] as described previously [53,54]. For each material five cylindrical samples (15 mm diameter, 1 mm thickness) were made. Water sorption (Equation (1)) and solubility (Equation (2)) were calculated according to following equations:

(1) $$W_{sp}=\frac{m_{2}-m_{3}}{V}$$

(2) $$W_{sl}=\frac{m_{1}-m_{3}}{V},$$

where: *W_sp_* is the water sorption, *W_sl_* is the water solubility, *m*_1_ is the initial constant mass (µg), *m*_2_ is the mass after seven days of water immersion (µg), *m*_3_ is the final constant mass (µg), *V* is the specimen volume (mm^3^).

#### 4.2.5. Microstructure Evaluation Based on Scanning Electron Microscopy

The materials microstructure was investigated with a High-Resolution Scanning Electron Microscope (HR-SEM) (FEI Nova NanoSEM 450, FEI, Hillsboro, OR, USA) equipped with a high-sensitivity Circular Backscatter (CBS) detector the for detection of backscattered electrons (BSE). The chemical composition was analyzed using an energy dispersive spectrometer (EDS, EDAX/AMETEK, Materials Analysis Division, Model Octane Super, Mahwah, NJ, USA). Prior to the analysis, the resin samples were coated with a 10-nm layer of gold.

#### 4.2.6. Statistical Analysis

Descriptive statistics were prepared, and the results were subjected to statistical analysis using Statistica version 13 software (StatSoft, Kraków, Poland). The distribution of continuous variables was tested using the Shapiro–Wilk Test of normality. Based on the results, the Kruskal–Wallis test with multiple comparisons of mean ranks or the one-way ANOVA with post hoc test (Fisher’s Least Significant Difference) were applied. The accepted level of significance was α = 0.05. Based on their distribution and homogeneity of variance, the results are given as either mean values with standard deviation (SD), or median values with quartile deviation (QT).

## 5. Conclusions

On the basis of this study result, the following conclusions can be drawn:

- Each aging protocol has some influence on the tested properties of Resin F, Flow-Art and Arkon;
- The mechanical properties of neat resin (Resin F) were more susceptible to aging protocols than the filled materials (Flow-Art, Arkon);
- The most aggressive aging protocol was Protocol 5 (0.1 M NaOH, 7 days, 60 °C);
- Protocol 4 (7500 thermocycles, 5/55 °C) was more aggressive than Protocol 3 (500 thermocycles, 5/55 °C) and it had a greater influence on materials with higher filler content (Arkon);
- When designing studies on dental materials, there is a need for accurate selection of the thermal and chemical factors acting during artificial aging.

## Figures and Tables

**Table 1:** The results of flexural strength (FS), diametral tensile strength (DTS) and hardness (HV) for tested materials after selected aging protocols. The results with the same assigned letter within the same material (Resin F, Flow-Art or Arkon) are significantly different (*p* ≤ 0.05).

Material	Aging Protocol	FS [MPa]	SD	FM [MPa]	QT	DTS [MPa]	SD */QT	HV [-]	SD */QT
Resin F	1	87.5 ^a,b,c,d^	9.1	1950	310	36.6 ^a^	8.3	16 ^a,b^	1
	2	68.1 ^a^	13.4	2130 ^a^	170	22.7 ^a^	3.3	15	2
	3	77.4 ^e,f^	5.8	1920	170	32.1	6.8	15 ^a^	1
	4	65.1 ^b,e^	15.8	1980	160	28.0	4.3	15	1
	5	72.5 ^c^	8.2	2160	510	34.9	15.7	15 ^b^	0
	6	62.7 ^d,f^	11.6	1790 ^a^	260	25.4	9.1	15	1
Flow-Art	1	97.0 ^a,f^	16.2	5750 ^a,b^	620	47.5	5.7	35 ^a,b,c^	1
	2	87.5 ^b^	10.9	5840 ^c,d^	440	40.0 ^a^	7.6	32 ^a^	4
	3	84.2 ^c^	15.6	4480 ^a,c^	440	44.3	6.8	31 ^b^	1
	4	84.3 ^d^	7.6	5720	320	45.6	13.3	34 ^d^	1
	5	58.4 ^a,b,c,d,e^	10.8	5250	2050	38.6 ^b^	6.8	29 ^c,d,e^	3
	6	78.2 ^e,f^	15.3	4090 ^b,d^	1050	48.6 ^a,b^	3.1	35 ^e^	1
Arkon	1	98.6 ^a,e,i^	9.7	9360	3130	44.4 ^a,f^	6.6 *	63 ^a,f,j^	2 *
	2	98.6 ^b,f,j^	9.6	9620 ^a^	960	40.6 ^b,g^	5.2 *	53 ^a,b,c,d,e^	3 *
	3	104.5 ^c,g,k^	5.8	7710	1980	52.6 ^a,b,c,d,e^	5.5 *	63 ^b,g,k^	1 *
	4	84.4 ^a,b,c,d^	6.3	9360	860	30.3 ^c,f,g,h,i^	5.4 *	63 ^c,h,l^	2 *
	5	53.0 ^d,e,f,g,h^	8.7	8180	3120	44.0 ^d,h^	6.5 *	48 ^d,f,g,h,i^	2 *
	6	79.8 ^h,i,j,k^	14.4	8300 ^a^	2000	44.7 ^e,i^	5.0 *	61 ^e,i,j,k,l^	2 *

**Protocol 1**—24 h, 37 °C, distilled water; **Protocol 2**—three months, 37 °C, artificial saliva; **Protocol 3**—500 cycles, 5 °C and 55 °C, water; **Protocol 4**—7200 cycles, 5 °C and 55 °C, water; **Protocol 5**—seven days, 60 °C, 0.1 M NaOH; **Protocol 6**—five days, 55 °C, water. Values are given as mean with standard deviation (SD) or median with quartile deviation (QT) based on distribution and homogeneity of variance; *—average value with the standard deviation.

**Table 2:** Mean sorption and solubility for Resin F, Flow-Art and Arkon materials with standard deviation (SD). Sorption and solubility were determined according to the ISO 4049 standard.

Material	Sorption (SD) [µg/mm^3^]	Solubility (SD) [µg/mm^3^]
**Resin F**	35.6 (0.8)	1.7 (0.6)
**Flow-Art**	21.6 (1.2)	1.2 (0.5)
**Arkon**	14.6 (0.5)	0.7 (0.2)

**Table 3:** Detailed information about selected materials used in the first stage of the study.

Material Name	Manufacturer	Type	Composition	Curing Time [S]
RESIN F	Arkona (Niemce, Poland)	Resin	Matrix: Bis-GMA, TEGDMA, UDMA, Bis-EMA, CQ: DMAEMA	20
FLOW-ART		Flowable composite	Matrix: Bis-GMA, TEGDMA, UDMA, Bis-EMA; CQ: DMAEMA Filler: Al-Ba-B-Si glass, Ba-Al-B-F-Si glass, pyrogenic silica (62 % wt.)	20
ARKON		Universal composite	Matrix: Bis-GMA, TEGDMA, UDMA, Bis-EMA; CQ: DMAEMA Filler: Al-Ba-B-Si glass, Ba-Al-B-F-Si glass, pyrogenic silica (78 % wt.)	20

**Table 4:** Preselected aging protocols.

Aging Protocol	Description
**1**	24 h, 37 °C, distilled water; standard method of sample conditioning (for example in ISO 4049—flexural strength [27])
**2**	Three months, 37 °C, artificial saliva; time needed to stabilize weight gain [51]
**3**	500 cycles, 5 °C and 55 °C, water; based on ISO/TS 11405:2015 [25]
**4**	7200 cycles, 5 °C and 55 °C, water; this corresponds to one year of clinical function, assuming that 20 such cycles may occur per day [52]. Previous studies have noted major deterioration before day 4 in resin cements (≈6000 cycles) [26]
**5**	One week, 60 °C, 0.1 M NaOH; this is considered to be a quick appropriate method to predict the durability of composites in vitro [23]
**6**	Five days, 55 °C, water; considered a viable alternative to thermal cycling [26]

## Data Availability

Data available in a publicly-accessible repository: Zenodo at: https://doi.org/10.5281/zenodo.6583563 (accessed on 12 January 2022).
